# Fabrication and characterization of efficient visible-NIR CdS/porous Si/p-Si photodetectors *via* laser-enhanced chemical bath deposition

**DOI:** 10.1039/d5ra08224b

**Published:** 2026-01-14

**Authors:** Raghad H. Mohsin, Hasan A. Hadi, Raid A. Ismail

**Affiliations:** a Department of Physics, College of Education, Mustansiriyah University Baghdad Iraq; b Department of Applied Science, University of Technology Baghdad Iraq raidismail@yahoo.com

## Abstract

The progress in preparation techniques of semiconducting thin film is a fundamental aspect of present-day electronics, addressing the increasing need for high-efficiency and cost-effective optoelectronic devices. The CdS-porous silicon (PSi) heterostructure has drawn significant attention for advanced technological and industrial applications, such as solar cells and photodetectors. Nevertheless, conventional chemical bath deposition (CBD) commonly produces CdS films with limited crystallinity, high defect density, and non-uniform morphology, which reduce device performance. Therefore, developing a simple, cost-effective technique to improve the structural quality of CdS without modifying the chemical route is highly desirable. This study presents a method that uses green diode laser illumination to improve the quality of the CdS films prepared by the CBD process. X-ray diffraction XRD investigations of the CdS film prepared with laser illumination reveal improved crystallinity compared with the as-deposited CdS film. Optical absorption measurements show that the optical energy gap of the CdS film increases from 2.38 eV to 2.61 eV when laser illumination is employed during film deposition. Scanning electron microscopy (SEM) studies confirm a reduction in the agglomeration of CdS particles when subjected to laser illumination, with the CdS particles being effectively incorporated inside the pores of PSi. The CdS-embedded PSi photodetector shows an increase in responsivity from 1.9 to 4.5 A W^−1^ at 480 nm when laser illumination is applied during deposition. Additionally, the rise and fall times of the photodetector fabricated with laser illumination are 159 ms and 208 ms, respectively. The results indicate improved spectral response and external quantum efficiency (EQE), enhanced detection capability, and reduced noise equivalent power (NEP), signifying greater device sensitivity to laser light.

## Abstract 2

High-performance vis-NIR CdS/porous Si/Si heterojunction photodetectors were fabricated using a low-cost one step laser-assisted chemical bath deposition technique.

## Graphical abstract

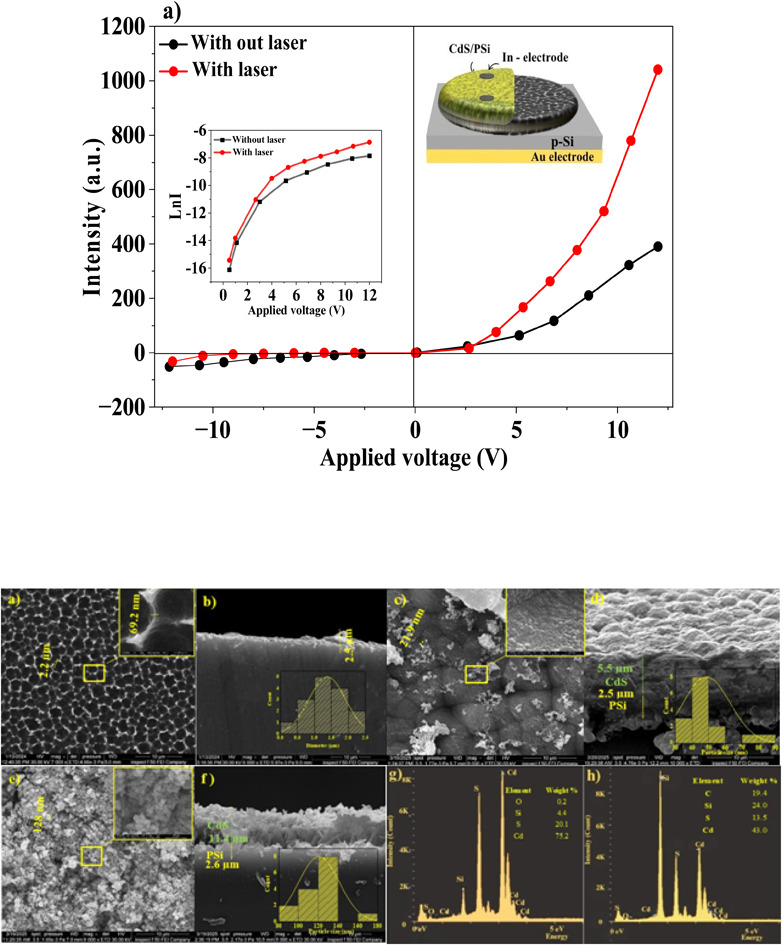

## 1. Introduction

Cadmium sulfide (CdS) is an attractive chalcogenide semiconductor, characterized by a direct optical energy gap of 2.25 to 2.42 eV at room temperature. As a crucial II–VI compound semiconductor for optoelectronic devices, CdS thin films are notable for their excellent optical and electrical properties.^1,2^ CdS is naturally obvious mostly as yellow and orange crystals, presenting in two phases: hexagonal and cubic crystal structures according to preparation method and conditions.^3^ They have been prepared using a variety of methods such as sputtering,^4^ spray pyrolysis,^5^ vacuum evaporation,^6^ pulsed laser deposition,^7^ and chemical bath deposition (CBD). It is well known that chemical bath deposition (CBD) is a low-temperature processing technique, a low-cost, large-area deposition method, and a straightforward process that does not need any complex equipment, such as a vacuum. It is an ancient technique for film deposition that produces uniform films with excellent adhesion to the substrate. Since the 1960s, it has been used in the deposition of PbS semiconductor thin films.^8,9^ A window layer in CdS/CdTe and Cu(In,Ga)Se_2_ (CIGS) solar cells is one of the significant uses for CdS thin film.^10,11^ Sami *et al.*^12^ used the spin coating technique to deposit CdS thin films on glass substrates with different Cd and S concentrations. Ahmed *et al.*^13^ investigate the impact of different deposition durations on the characteristics of CdS films fabricated using the CBD method. Experimental results indicate that all formed films have a crystalline structure, with a structural transition from cubic to hexagonal occurring when deposition time extends from 60 to 120 minutes. Kakhaki *et al.*^14^ studied the photodetection characteristics of CdS thin films prepared using the CBD technique at varying concentrations of cadmium chloride. They showed that the sample Cd (0.008 M) had a photosensitivity more than 200 times that of the sample prepared at Cd (0.08 M). Eesa *et al.*^15^ utilized chemically synthesized CdS nanopowder, which was spin-coated onto silicon and porous substrates, to produce a cadmium sulfide photodetector (PD). The photodetector's responsivity was 0.03 A W^−1^.

Li *et al.*^16^ studied the electrical properties of a CdS/Si nano heterostructure array fabricated by the CBD route. Srinivasarao *et al.*^17^ fabricated an effective optoelectronic device *via* deposition of Au nanoparticles on the CdS thin films prepared by pulsed laser deposition. Porous silicon (PSi) has emerged as a viable material for photosensing applications because of low power consumption, a tunable energy gap, high bias voltage operation, a large surface area, high light trapping, improved charge carrier transport, a small dark current, and compatibility with silicon-based technology. The photosensitivity of PSi is contingent upon the morphological attributes of the pores, including pore diameter and uniform surface regularity, as well as the thickness and porosity of the porous layer.^18,19^ Vertical nanostructure arrays on Si surfaces, including nanowires, nanocones, nanopillars, nanotubes, and nanoheles, have garnered a lot of interest due to their remarkable physical properties, which come from the combination of convex dimensions and high surface adsorption compared to bulk materials. These arrays are attractive for optoelectronic applications because they may be utilized as models to form radial heterojunctions.^20–22^ In comparison to the crystalline silicon surface, the porous structure of silicon may have a higher surface-to-volume ratio since it typically has a thickness of several microns.^23^

Many methods are used to prepare porous silicon; one of the most interesting techniques is electrochemical etching. The CdS film has been used in many types of heterojunction photodetectors, such as ZnO/CdS,^24^ PSi/CdS,^25^*etc.* Because the mismatch in lattice constants of Si and CdS is quite small, it was able to manufacture a high-performance CdS/Si heterojunction. Based on the information presented, the CdS/PSi photodetector offers a number of advantages, such as fast rise and fall times, low dark current, high bias operation, low cost, minimal integration issues with modern silicon-based electrical devices, and high responsivity in the visible and near-infrared ranges.^26,27^

Nevertheless, despite these benefits, limited crystallinity, irregular morphology, and flaws are frequently produced by the traditional chemical bath deposition (CBD) of CdS films, which might lower the effectiveness and dependability of photodetectors. Furthermore, few research have looked at straightforward, inexpensive ways to enhance the structural and optoelectronic characteristics of CdS films without changing the chemical deposition procedure.

The present study proposes a route using green diode laser illumination prior to CBD to enhance the growth properties of CdS films. The effect of laser illumination on the structural, morphological, and electrical properties of the CdS films is systematically investigated. Additionally, the figures of merit of CdS/PSi photodetectors fabricated with and without laser illumination are evaluated to demonstrate the potential improvements achieved using this approach.

## 2. Experimental work

### 2.1. Preparation of porous silicon

**Fig. 1:**
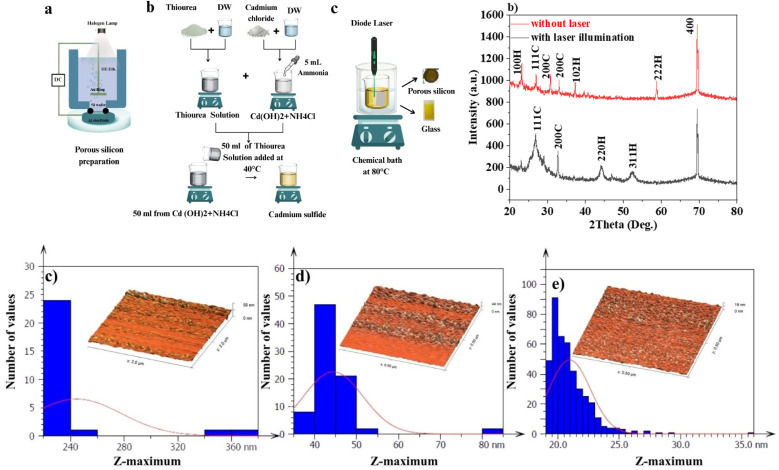
(a) Preparation of porous silicon and deposition of CdS film, (b) XRD pattern of the CdS films prepared by CBD and LACBD, (c) 3D AFM images and GSD of PSi, (d) 3D AFM image and GSD of CdS film deposited by CBD, and (e) 3D AFM image and GSD of CdS film prepared by LACBD.

In this study, the electrochemical etching method was used to prepare the PSi layer. A 500 µm-thick, p-type (100) silicon wafer with an electrical resistance of 10 Ω cm was used. The silicon wafer is cut into pieces, each having an area of 1 cm^2^. The samples were subsequently cleaned in an ultrasonic bath for five minutes using ethanol, acetone, and distilled water. Subsequently, we used hot air to desiccate the samples. The porous silicon was synthesized *via* a homemade electrochemical etching apparatus, as shown in Fig. 1(a). The current density and etching time were selected to be 8 minutes and 10 mA cm^−2^, respectively; this condition provides a thin layer and a low-porosity surface.

### 2.2. Deposition of CdS thin film

The Laser-Assisted Chemical Bath Deposition (LACBD) method was used to prepare the CdS thin films. CdS thin films were deposited on glass and porous silicon substrates in 90 minutes. The concentrations of cadmium chloride (CdCl_2_·2H_2_O) and thiourea (SC(NH_2_)_2_) were maintained at 0.1 M and 0.2 M, respectively, for each experiment. The chemical bath was prepared in a glass beaker with 100 ml of deionized (DI) water that was kept at 80 °C and constantly stirred. The substrates used were glass slides with a dimension of 25 × 25 × 1 mm. In the process of preparation, the first step is cleansing them with liquid soap and doing a thorough rinse with tap water. After that, they were soaked in methanol, followed by a washing using liquid soap and a thorough rinse with tap water. Thereafter, they were placed in methanol, followed by a washing using deionized (DI) water, and finally, the samples were dried with a stream of nitrogen before the deposition procedure. In order to deposit a CdS film, a solution was prepared by combining cadmium sulfate and 5 ml of aqueous ammonia in deionized water at room temperature. Subsequently, thiourea was added to the solution at 50 °C. Following, 5 ml of aqueous ammonia was added. The volume of the final solution was approximately 100 ml. Throughout the chemical bath deposition of CdS films, ammonia can serve as a complexing agent by binding Cd^2+^ ions that controls the release rate of cadmium and prevents rapid precipitation in solution. This in turns help achieve uniform nucleation and smooth film growth. The pH of the solution, which is usually changed with ammonia, controls the nucleation rate and the availability of sulfide ions, which affects the adherence, morphology, and crystallinity of the formed CdS films. Therefore, deposition of high-quality films with optimal structural and optical properties requires careful adjustment of pH and ammonia concentration. The chemical bath procedure was carried out for 90 min deposition time at 80 °C. At the same time, a continuous diode laser of *λ* = 532 nm with an output power density of 16 mW cm^−2^ was used to illuminate the substrate during film deposition. To avoid film deposition on the reverse surfaces of the glass substrates and remove the hydroxyl compound formed during the chemical reaction, we used a wax coating. Fig. 1(a) illustrates the schematic diagram of LACBD system. The CdS thin films were grown by the hydrolysis of thiourea in a solution of acid containing a cadmium salt and ammonia, using the following chemical reactions:^28,29^

1 CdCl_2_ + 4NH_3_ → Cd(NH_3_)_4_Cl_2_

2 [Cd(NH_3_)_4_]^2+^ → Cd^2+^ + 4NH_3_

3 Cd^2+^ + 2OH^−^ → Cd(OH)_2_ (formation of cadmium hydroxide cluster)

4 CS(NH_3_)_2_ + OH^−^ → CH_3_N_3_ + H_2_O + S^2−^ (thiourea is broken down by alkaline water)

5 [Cd(NH_3_)_4_]^2+^ + S^2−^ → CdS + NH_3_ (deposition)

### 2.3. Characteristics

The structural characteristics of the CdS films and porous silicon were analyzed using the AERIS-XRD X-ray diffractometer from PANalytical. The film's topography was examined with an atomic force microscope (AFM) model TT-2. The morphology of the films and porous silicon was examined using field emission scanning electron microscopy (FESEM) (Inspect F 50-FEI). A Raman microscope (Bruker Sentera, Germany) with a 532 nm and 58 nm laser wavelength was used to investigate the vibrational modes of the CdS layer. The optical absorbance of the film was evaluated using a UV-vis spectrophotometer (Jasco V670/Japan).

### 2.4. Fabrication of CdS/PSi heterojunction

CBD and LACBD were used to deposit the CdS film on porous silicon to fabricate a CdS/PSi photodetector. Using thermal evaporation and DC sputtering systems, ohmic connections were formed by evaporating indium on CdS film and gold on the backside of p-type silicon through a mask.

The net photosensitive area of the photodetector was 0.282 cm^2^. Ohmic contacts on the CdS/pSi/Si structure were formed by depositing indium (In) on the CdS film using the thermal resistive technique under a vacuum pressure of 10^−7^ torr. Aluminum (Al) films were also deposited on the backside of the silicon substrate to establish the rear ohmic contact with thickness of 300 nm.

After making the ohmic contacts, connections to the circuit are performed by making wiring from the photodetector using silver paste. The figures of merit, including current–voltage, responsivity, detectivity, and rise and fall times, were investigated using the photodetector evaluation system PES. It consists of a 100 W halogen lamp with a parabolic reflector, monochromator, electrometer, DC power supply, beam splitter, silicon power meter, laser diode, and digital CRO.

## 3. Results and discussion

Fig. 1(b) shows the XRD patterns of CdS films deposited on porous silicon substrates using CBD and LACBD methods. This figure confirms that the CdS films were polycrystalline. Seven peaks were detected for CdS film prepared by CBD at 2*θ* = 23.24°, 26.96°, 30.76°, 33.02°, 37.28°, 58.78°, and 69.5°. These peaks corresponded to the (002), (111), (200), (200), (102), (222), and (400) planes, respectively. The first six XRD peaks are matched with cubic and hexagonal CdS according to JCPDs # 89-0440, 00-021-0829, and JCPDs # 01-075-1545.^30^ The seventh strong peak belonged to the porous silicon substrate. While the XRD pattern of the CdS film deposited on a porous silicon substrate with laser illumination reveals the presence of four peaks located at 2*θ* = 26.8°, 44.2°, 52.3°, and 69.2°, corresponding to the (111), (220), (311), and (400) planes, respectively.

As shown in Fig. 1(b), it is obvious that the XRD pattern of the film deposited with laser exhibits broader peaks with lower intensities, suggesting the formation of nanostructured CdS film. The broadening of the X-ray peak is due to a reduction in crystallite size of the CdS film. The average crystallite size of the CdS films was estimated using Scherrer equation^31^6
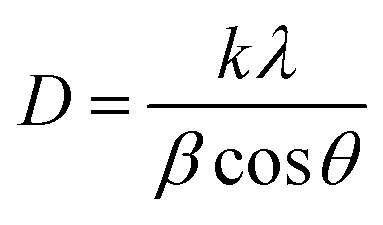
where *λ* is the Cu Kα X-ray wavelength (*λ* = 0.15406 nm) and *β* is the full width at half maximum FWHM of the peak.

The lattice strain (*ε*) and dislocation density (*δ*) of produced in CdS films during growth and nucleation processes were calculated from XRD analysis using the following relationships:^32^7
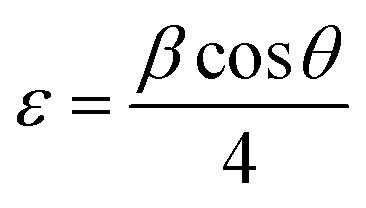
8
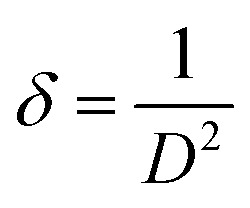


As shown in Table 1, the crystallite size of the CdS film prepared by LACBD is smaller than that of the CdS film deposited by CBD. Since the average crystallite size decreases from 55 to 18 nm as the film illuminated by laser during deposition. As shown in Fig. 1(b), the XRD patterns of the CdS films prepared with laser illumination formation of fewer and broader peaks compared with the conventionally deposited films. We attribute this broadening to the formation of nanostructured CdS with small crystallite size, as some particles are embedded within the pores of the silicon substrate. Importantly, no new peaks or peak shifts were observed, indicating that the phase purity is preserved. The decrease in the peak intensity for certain planes may also suggest a slight influence of the porous substrate on crystal growth, but no significant preferred orientation is observed. Since nanostructure can improve carrier collection and light absorption, these structural characteristics are compatible with the enhanced optical and electrical capabilities seen for the laser-assisted CdS films.

**Table 1:** Lists the crystallite size, strain, and dislocation of the CdS films deposited by CBD and LACBD

Laser effect	2*θ* (deg.)	FWHM (deg.)	Average crystallite size	*hkl*	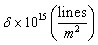	*ε* × 10^−3^
Without laser	23.21	0.1692	55.17	100	0.435	0.723
	26.96	0.1446		111	0.313	0.613
	30.76	0.2148		200	0.680	0.903
	33.02	0.1080		200	0.169	1.5
	37.28	0.1508		102	0.323	0.62
	58.78	0.3504		222	0.147	1.332
With laser	26.8	0.3835	18.2	111	0.220	1.627
	32.7	0.2305		200	0.775	0.965
	44.2	0.9327		220	0.118	3.770
	52.3	1.3241		311	0.223	5.185

Furthermore, the film deposited with LACBD route showed a decrease in dislocation density and lattice strain. The nucleation rate on the substrate surface is increased by laser illumination, which can be attributed to localized heating and enhanced ion mobility in the reaction zone. Consequently, each crystallite's development is slowed down as more nuclei arise and vie for the available growth species. Moreover, the surface tension and slight temperature gradients induced by continuous laser irradiation may decrease grain coalescence and disturb long-range crystal ordering.

In order to examine the topography of porous silicon layer and the impact of laser illumination on the topography of CdS thin films deposited on the porous silicon surface, atomic force microscopy (AFM) was used. Fig. 1(c–e) displays the 3D AFM images along with histograms that illustrate the surface topography of porous silicon as prepared and the CdS films that were deposited on a porous silicon substrate without and with laser irradiation.

Fig. 1(c) shows 3D AFM images of porous silicon surface. The root mean square of surface roughness RMS of porous silicon was 2.1 nm. The histogram of average grain size shows a high distribution of PSi calculated in the range of 220 and 400 nm. Fig. 1(d and e) showed that the whole surface of the porous silicon was covered by the CdS thin film layer as well as incorporated with the grains of the CdS film. Some film grains are on the walls between the pores, but most are in the pores. When compared to a sample deposited without laser irradiation (Fig. 1(d)), the one formed with laser illumination exhibits a more uniform dispersion of CdS grains. Fig. 1(e). According to the data shown in Table 2, the RMS of the CdS film deposited without and with laser irradiation were 1.96 nm and 3.99 nm, respectively. The increase in the surface roughness is probably due to the enhanced surface nucleation and particle coalescence, since an increased temperature due to laser illumination helps fast nucleation, leading to islanded or columnar microstructures.

**Table 2:** AFM analysis of porous silicon and CdS films

Sample	RMS (nm)	Roughness average *S*_a_ (nm)
PSi	2.186	1.498
CdS/PSi prepared with CBD	1.969	1.545
CdS/PSi prepared with LACBD	3.995	2.863

For film deposited by LACBD, the laser beam continually and locally increases the temperature of the film to increase supersaturation. This finding is advantageous for selective nucleation, as it results in the development of tall grains with a variety of irregular heights and shapes. Conversely, chemical bath deposition (CBD) exhibits a non-uniform surface and is dependent on slower, diffusion-limited deposition processes. The increased roughness of the film is beneficial for photodetection applications, since it may enhance light trapping. In films deposited by LACBD, the laser beam continually and locally increases the temperature of the film to increase supersaturation. On the other hand, the increased roughness of the film is necessary for photodetection applications, since it may enhance light trapping.

**Fig. 2:**
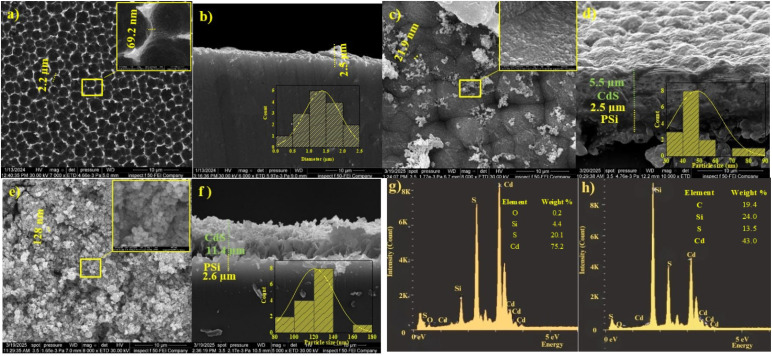
FESEM images and EDX spectra showing the surface and compositional structures of porous silicon (PSi) layers and CdS/PSi films prepared with and without laser illumination (a) top view of the PSi surface with an inset image at higher magnification showing the pore distribution; (b) cross-section of the PSi layer with an inset image of a histogram showing the distribution of average pore diameters, (c) CdS/PSi film prepared without a laser and an inset image at higher magnification; (d) cross-section of a CdS/PSi film prepared without a laser, with an inset image of a histogram showing the distribution of particle diameters in the film, (e) CdS/PSi film prepared with a laser and an inset image at higher magnification, (f) cross-section of the CdS/PSi film prepared in the presence of a laser, with an inset image showing the CdS layer thickness and a histogram of the particle size distribution, (g and h) EDX spectra of the CdS/PSi samples prepared without and with a laser respectively.

The FESEM images with different magnifications of porous silicon and CdS films deposited on porous silicon with and without laser illumination are depicted in Fig. 2(a, c and e). Fig. 2(a) displays the SEM image of the surface PSi sample etched for 8 minutes with a current density of 10 mA cm^−2^. The surface is characterized by semi-circular pores, notable for their uniform distribution and high density of pores. The average pore size and porosity were 1.3 nm and 51% nm, respectively, the thickness of porous silicon layer was 2.5 µm, as shown in Fig. 2(b).

Fig. 2(c) shows the FESEM image of CdS film deposited on PSi without laser illumination, which confirms the formation of spherical CdS nanoparticle. As shown, the CdS nanoparticles do not distributed uniformly and embedding inside the pores matrix. The inadequate coverage and irregular particle distribution, since the pores of the PSi matrix are only partly filled by the CdS nanoparticles which leads to scatter the light and deteriorates the photodetection properties of the CdS/PSi photodetector. Fig. 2(d) is the FESEM image of the CdS-PSi cross-section, which reveals the film thickness was 5.5 µm. The FESEM image in Fig. 2(e) illustrates that the CdS nanoparticles prepared by LACBD that embedding PSi are much finer, more uniform, and covered the whole porous silicon surface. As seen in Fig. 2(e), the FESEM image of the cross-section verifies that the film thickness was 11.4 nm. CdS film it looks less thick, more compact and has fewer voids, suggesting that laser illumination improves precursor decomposition and nucleation, resulting in finer particles that more successfully enter the pores.

As shown in Fig. 2(g and h), the energy dispersive X-ray (EDX) spectra of CdS films grown on porous silicon substrates. The main detected elements were Cd, S, and Si, which are the main elements of the CdS film and porous silicon. The film deposited without laser illumination, as depicted in Fig. 2(g), has atomic percentages of 75.21 and 20.1 for Cd and S elements, respectively. While, the CdS film prepared with laser illumination exhibits an atomic percentage of 4.9 and 10, for Cd and S, respectively, as shown in Fig. 2(h). The low percentage of Cd may be due to the high content of silicon from the substrate. These results confirm that the stoichiometry of the CdS film was improved when the laser beam illumination used during film deposition. Based on EDX analysis, the atomic perecntage [Cd]/[S] ratio was found to be 0.9 for CdS film deposited with laser illumination, which is close to stoichiometric CdS film with ratio of 1. Whereas the atomic percentage ratio of [Cd]/[S] for the film prepared without a laser was 1.06.

**Fig. 3:**
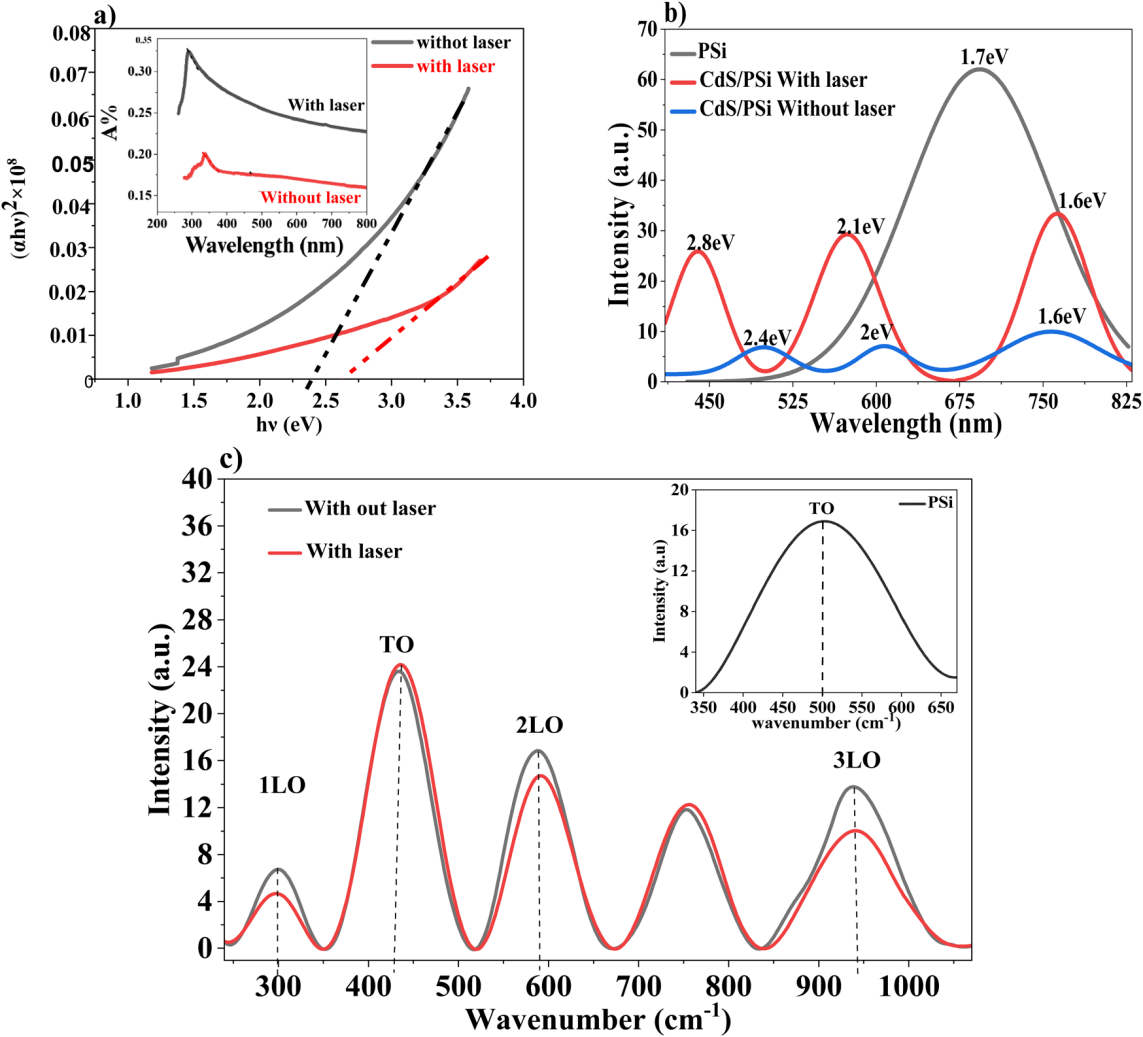
Optical and structural properties of CdS film. (a) Optical bandgap of CdS thin films prepared with (black) and without laser (red). The inset shows the absorption spectra of the film without a laser (black line) and with a laser (red line). (b) Photoluminescence (PL) spectrum of PSi layer as-prepared (black line), CdS/PSi films prepared without (blue line) and with laser (red line). (c) Raman spectra of CdS/PSi films prepared without a laser (black line) and with a laser (red line), the inset of (c) shows the Raman spectrum of PSi.

The inset of Fig. 3(a) shows the impact of laser illumination on the optical absorbance of CdS film deposited on the glass substrates without and with laser illumination. The absorbance of the CdS films, both deposited without and with laser illumination, decreases as wavelength increases until reaching 520 nm and 480 nm (absorption edges), respectively, after which it saturate. The absorption of the CdS film deposited without laser illumination was higher than that of the CdS film deposited with laser illumination as a result of the scattering caused by the irregular, large particulates that covered the film. Conversely, the CdS film that is deposited using a laser illumination exhibits a lower absorbance as a result of the finer film surface, which is a result of the reduced surface irregularity. The laser illumination leads to a fast nucleation process, resulting in the growth of nanoparticles that are smaller, denser, and more uniform. The optical energy gap (*E*_g_) of the CdS film for direct electronic transitions was determined using the Tauc relation equation:^33^

9 (*αhν*)^2^ = *A*(*hν* − *E*_g_)

here *A* is a constant that varies with the type of material, and *α* is the absorption coefficient that is found from the optical absorbance spectra as a function of the photon energy (*hν*). Fig. 3(a) shows a plot of the variation of (*αhν*)^2^ with photon energy. The optical energy gap is found by extrapolating the linear part of the curve of Fig. 3(a) to the photon energy axis.

The particle size of the CdS was estimated from energy gap shift (Δ*E*_g_ = 0.23 eV) using Brus equation:10
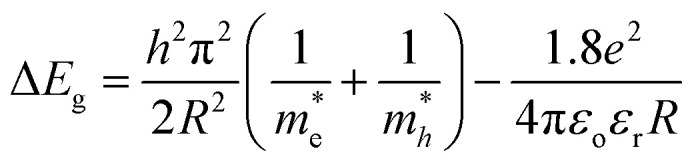
where *R* is the particle size of the CdS, 
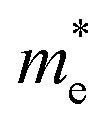
 is the effective electron and hole masses 
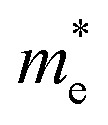
 and 
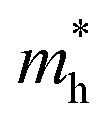
 of the CdS are 0.19 *m*_0_ and 0.8 *m*_0_, respectively, the relative dielectric constant of CdS = 5.7. After substituting all the values and constants into eqn (10), the particle size of the CdS film was calculated to be 12.2 nm. This value is smaller than that obtained from XRD data, which can be attributed to stress induced by the porous silicon substrate.

Fig. 3(b) shows the photoluminescence spectra for three different structures: a CdS/PSi sample with laser applied during chemical bath deposition (CBD), a cadmium sulfide (CdS) layer on PSi without laser, and porous silicon (PSi/p-Si). A very modest bandgap and primarily direct transitions are indicated by the broad emission peaks in the near red region of the PSi sample (around 1.6 eV or 690 nm), which are ascribed to transitions within defect centers and surface bonding (Si–O and Si–H). In the blue visible region, the sample prepared without a laser exhibits three main peaks at energies of roughly 1.6 eV (758.2 nm), 2.0 eV (606.4 nm), and 2.4 eV (498.4 nm). The origin of the peaks at 606.4 nm and 758.2 nm is sulfur vacancies and deep-level defects and surface states, respectively. In contrast, the sample prepared with a laser exhibits similar peaks, but it is obviously shifted towards higher energies (blue shift), found at roughly 1.6 eV (763.9 nm), 2.1 eV (573.2 nm), and 2.8 eV (439.1 nm). The results show a regular blue shift throughout the spectrum, with all peaks shifting toward higher energies from (100–400 meV) and a drop in wavelengths between 763.9 and 439.1 nm. The results demonstrate a blue shift in nanoparticles, which is linked to the laser's effective role in the chemical preparation process, enhancing nucleation growth while minimizing nanoparticle diameters. This results in improved quantum confinement effects within silicon pores. Additionally, the laser photothermal effect enhances crystallinity and reduces internal defects, significantly increasing the efficiency of direct radiative transformations and the intensity of photoluminescence (PL) peaks in samples treated with the laser.

Fig. 3(c) indicates the Raman spectra of PSi were recorded between 300 and 1200 cm^−1^ using an excitation wavelength of 523 nm. The Raman peak for untreated PSi is seen at 503 cm^−1^. This peak signifies the vibrational modes inherent in the porous silicon structure.

The technique of Raman spectroscopy, which is helpful in analyzing the microstructure of crystalline materials, the CdS microspheres were examined. II–VI compound semiconductors are recognized to crystallize in both the cubic zinc blende and hexagonal wurtzite structures. This confirms that one of the most remarkable features of the Raman spectra of CdS is the outstanding overtone series of the longitudinal optical phonons. As seen in Fig. 3(c), at 300, 600, and 900 cm^−1^, three distinct peaks that correspond to LO phonon scattering. These peaks are referred to as 1LO (first order), 2LO (second order), and 3LO (third order), respectively. Originated from phonon vibration.^34^ No shift in Raman peaks for CdS films deposited without laser (black curve) and with laser illumination (red curve)was noticed for both 1LO. 2LO, and 3LO. Enhanced crystallinity and higher phonon–photon interaction are both characteristics of the peaks that are much sharper and more intense. This suggests that the material has a crystal structure that is well defined, which makes it easier for phonons and photons to interact with one another in a more efficient manner. This may be attributed to the sharpness and intensity of the peaks that have been detected.

With the influence of the laser radiation, the Raman shift peaks of the CdS film were formed. This improvement may be the consequence of stress effects at the interface between the film and the substrate, reduced crystallite size dispersion, or the activation of phonon surface modes in nanoscale domains.

**Fig. 4:**
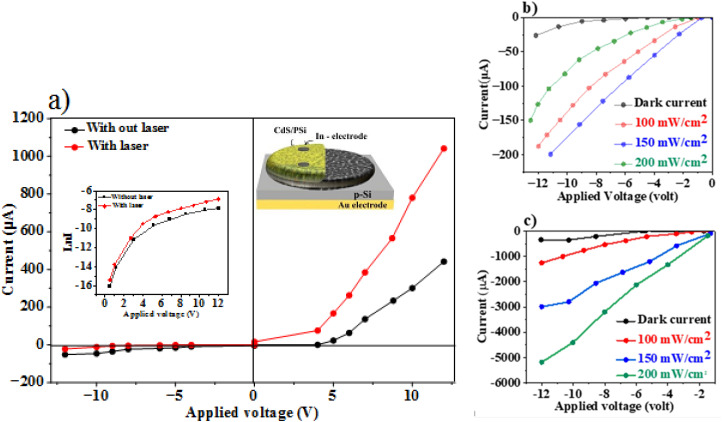
(a) The profile of the *I*–*V* characteristics, inset semi-logarithmic *I*–*V* plot and a cross-sectional schematic design of the CdS/PSi photodetector, (b) illuminated *I*–*V* characteristics of CdS/PSi photodetector prepared with laser, (c) illuminated *I*–*V* characteristics of CdS/PSi photodetector prepared without laser illumination.

Fig. 4(a) displays dark current–voltage measurements for a CdS/PSi/p-Si heterojunction photodetector made using electrochemical etching and chemical deposition (CBD) methods, with and without a laser illumination. The saturation current was calculated from the forward ln (*I*–*V*) curve as shown in the inset Fig. 4(a). The figure shows the *I*–*V* characteristics of the CdS/PSi/p-Si junction, which was examined in both forward and reverse directions with a bias voltage ranging from −12 to +12.

The rectification factor (*R*_F_) was estimated and it found to be 150 and 380 at 10 V for the heterojunctions prepared without laser and with laser illumination, respectively. The current flow mechanism of these photodetectors is described using thermionic emission theory. Saturation current, barrier height, and ideality factor of CdS/PSi/p-Si were determined using the thermionic emission theory. CdS/PSi/p-Si ideality factor (*n*) was obtained using the following diode equation.^33^11
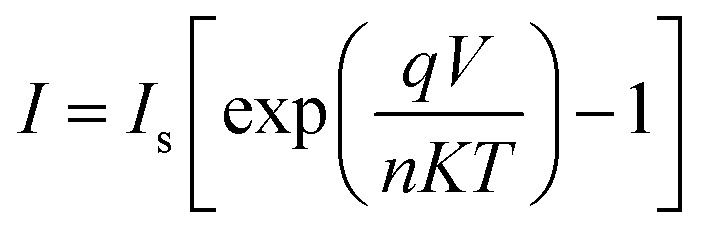
where *I*_s_ is the reverse saturation current. The semi-logarithmic plots of ln (*I*) *vs. V* were analyzed in order to determine the ideality factor *n* and saturation current using the exponential regions slope in accordance with simple diode equation.^35^Table 3 shows that the values of *I*_s_ of the CdS/PSi/p-Si photodetectors have decreased from 1 × 10^−7^ to 1.3 × 10^−8^ A, and the ideality factor *n* has decreased from 1.38 to 1.35 with laser illumination, indicating a reduction in recombination and traps. As depicted in Table 3, the laser illumination leads to an improvement in the RF values.^26^

**Table 3:** Saturation current, ideality factor, and rectification factor of CdS/PSi/p-Si photodetectors

Preparation condition	*I*_s_ (A)	*n*	*R*_F_
Without laser	1.3 × 10^−7^	1.38	150
With laser	1 × 10^−8^	1.35	380

Analyzing a heterojunction's current–voltage (*I*–*V*) characteristics under illumination is essential to comprehending photocurrent fabrication, which is an essential part of optoelectronic devices and optical signal conversion. The internal electric field is strengthened by increasing the reverse bias voltage, which makes it easy to separate electron and hole pairs. Photocurrent increases with light intensity without saturation, demonstrating the photodetectors' linearity and suitability for photonic applications. Fig. 4(b and c) shows the illuminated *I*–*V* characteristics of CdS/PSi/p-Si heterojunction photodetector fabricated with laser and without laser respectively. The photocurrent of the detector increased upon illumination due to the generation of electron–hole pairs within the depletion region. The device fabricated with laser illumination exhibited a higher photocurrent than that fabricated without laser assistance. This enhancement is attributed to the improved carrier transport efficiency and the larger effective surface area of the CdS film. As shown in Fig. 4(b), the CdS/PSi/p-Si heterojunction photodetector demonstrates excellent linearity and efficient separation of photogenerated carriers. The photocurrent increases proportionally with the incident light power density without reaching saturation, confirming its stable and efficient photoresponse. To investigate how laser illumination affecting a CdS/PSi/p-Si photodetector's performance, the spectrum responsivity *R*_*λ*_ was determined using the following formula, which is the ratio of the produced photocurrent to the incident light power^33^12
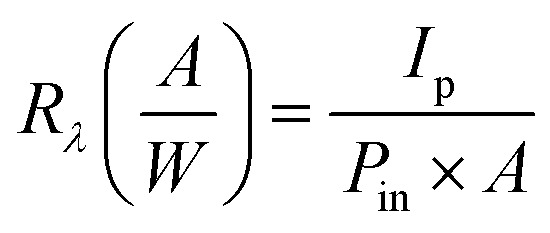
where: *R*_*λ*_ the spectrum responsivity (A W^−1^), where *I*_p_ is photocurrent (A), *P*_in_ is incident power density (W cm^−2^), and *A* is active area (0.2826 cm^2^).

**Fig. 5:**
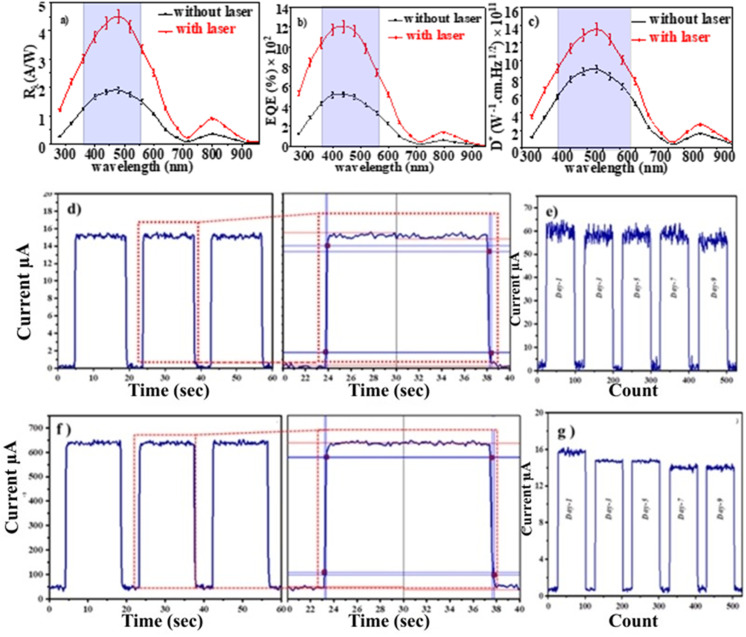
Figure-of-merits of CdS/PSi photodetectors, including (a) responsivity (*R*_*λ*_), (b) external quantum efficiency (EQE), and (c) specific detectivity (D*). The time-dependence characteristics and stability profiles of these photodetectors at 3 V bias voltage are shown in (d) and (e) without laser, in (f) and (g) with laser of intensity of 10 mW cm^−2^.

Two response peaks are shown in Fig. 5(a) for photodetectors made with and without laser illumination. The first peak is centered at 480 nm, while the second peak is at 800 nm. Compared to the CdS film formed without laser illumination, the laser-made film demonstrated a high responsivity. The photodetectors fabricated with and without laser illumination have responses of 1.9 and 4.5 A W^−1^ at 480 nm and 0.3 and 0.9 A W^−1^ at 800 nm, respectively.

The absorption edge of CdS film is the origin of the first response peak, while the absorption edge of silicon is the cause of the second response peak. The increased responsivity of the photodetector produced with laser illumination is ascribed to the expansion of the depletion region in addition to the superior optical transmittance of the CdS film resulting from light trapping. This result indicates that the generation of more electron–hole pairs in the depletion region, together with an increased diffusion length, subsequently enhances the responsivity of the photodetector. Conversely, the responsivity in the visible spectrum is greater than that in the near-infrared range. This discovery may be elucidated by the fact that the porous layer is entirely depleted owing to its electrical resistance and can be regarded as a depletion region. The thickness of the depletion layer *X*_t_ in CdS-PSi-c-Si can be expressed as follows:

13 *X*_t_ = *X*_CdS-PSi_ + *X*_PSi-c-Si_

*X*_CdS-PSi_ denotes the depletion layer width of the CdS film-porous silicon junction, whereas *X*_PSi-c-Si_ represents the depletion layer width of the porous silicon–crystalline silicon substrate junction. Across both examined locations. The enhanced photoresponse noted in the visible spectrum (480 nm) is ascribed to significant optical absorption at the CdS/PSi interface. The diminished spectral response at 800 nm results from absorption phenomenon occurring at the p-Si/PSi interface, particularly at the p-Si substrate.

The external quantum efficiency (EQE) is the ratio of photogenerated electrons to incident photons. It can be calculated using the following equation:^46^14
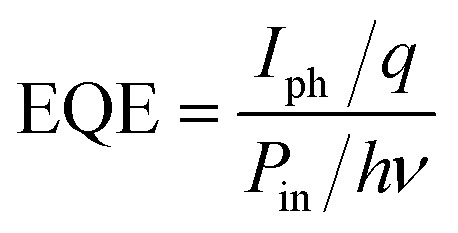


*P*_in_ represents the incident optical power (*W*), while *hv* denotes photon energy (*J*). Fig. 5(b) illustrates the external quantum efficiency (EQE) of CdS/PSi/p-Si photodetectors fabricated with and without laser illumination as a function of wavelength. The peak external quantum efficiency was recorded in the visible spectrum at 480 nm. Upon the application of laser illumination, the external quantum efficiency (EQE) escalated from 1200% to 500% at 480 nm, attributable to the augmented concentration of photogenerated carriers resulting from light trapping and the expansion of the depletion layer thickness. The elevated EQE value is primarily ascribed to the porous silicon layer, which functions as a depletion area that amplifies the internal electric field and therefore boosts the photocurrent. At extended wavelengths, the external quantum efficiency (EQE) was seen to decrease due to absorption occurring outside the depletion area inside the silicon substrate. The high value of EQE can be ascribed to trap-assisted mechanisms TAMs occurred in CdS/PSi heterostructures. Actually, TAMs play a important role in improvement the charge carrier transport. The porous silicon substrate introduces a high density of surface states and structural defects that can act as traps for electron or hole charge carrier. When CdS film is deposited on PSi, photogenerated carriers can be temporarily captured by these trap states, slowing their recombination and effectively increasing the carrier lifetime.

The specific detectivity (D*) is one of the most important parameters of a photodetector, as it represents the ability to detect weak optical signals. D* can be estimated using the following relation:^36^15
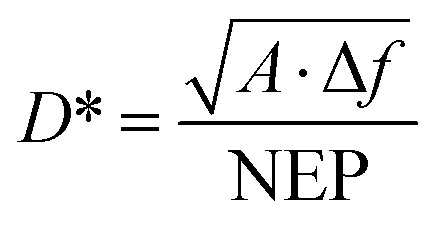
where Δ*f* denotes the bandwidth (Hz), and NEP represents the noise equivalent power (W). Fig. 5(c) illustrates the graph of D* as a function of wavelength for photodetectors fabricated with and without laser illumination. The specific detectivity of the CdS/PSi photodetector was 9 × 10^11^ Jones at 480 nm, while the CdS/PSi/Si photodetector, produced using laser illumination, attained a detectivity of 13 × 10^11^ Jones at the same wavelength.

At a wavelength of 480 nm, the NEP decreased from 5.9 × 10^−13^ to 4.0 × 10^−13^
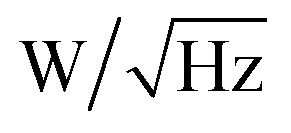
, indicating enhanced sensitivity as less optical power is needed to produce a signal matching its noise. While at 800 nm the improvement is less pronounced, there is a slight enhancement in D* and a decrease in NEP, suggesting improved response/noise efficiency under laser conditions.

Table 4 shows summarize the figures of merit of the Cds/PSi/p-Si photodetector. This improvement is due to the decrease in the leakage current and the substantial rise in the responsivity of the photodetector under effect of laser illumination.^37,38^

**Table 4:** Summarize the figures of merit of the CdS/PSi/p-Si photodetector

Preparation condition	Spectral responsivity *R*_*λ*_ (A W^−1^)		EQE (%)		Specific detectivity D* (Jones)		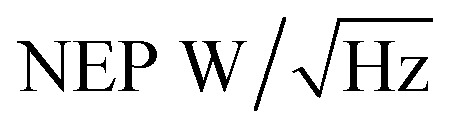	
	480 nm	800 nm	480 nm	800 nm	480 nm	800 nm	480 nm	800 nm
Without laser	1.9	0.3	5	0.6	9 × 10^11^	1.7 × 10^11^	5.9 × 10^−13^	3.1 × 10^−12^
With laser	4.5	0.9	12	1.4	1.3 × 10^12^	2 × 10^11^	4.0 × 10^−13^	2.6 × 10^−12^

The CdS/PSi/p-Si photodetector's dynamic photoresponse is shown in Fig. 5(d). The current increased from the dark state at rise time of 159 ms and its fall time 208 ms.

\The return of electrons and holes to equilibrium is somewhat slow due to locked energy levels (trap states) or recombination at the interface, as shown by the fall time 0.159 s, which is a little longer than the rise time. The response time was lower with a laser present during preparation than without one, as seen in Fig. 5(f); the rise time was 0.163 seconds and the recovery time was 0.303 seconds. This implies that by fortifying the CdS nanostructure, the laser enhanced transport electron–hole pair separation, and decreased trap states. Increased by the samples almost symmetrical and rapid light response, as seen by the recovery values and close rise. Using three daily automatic on–off cycles (6 minutes each), a 15-second pulse width, 10 mW cm^−2^ optical power, and at 3 V bias voltage, the stability of the photodetectors was confirmed. As shown in Fig. 5(e and g). The optical devices maintained constant operation without interruption, showing stable photodetector performance.

A comparison of the CdS/PSi photodetector fabricated by LACBD with other CdS/Si fabricated by other techniques is depicted in Table 5.

**Table 5:** Figures of merit of fabricated CdS/PSi compared to those for other fabricated CdS/Si photodetector

Fabrication technique	Substrate characteristics	CdS characteristic, particle (shape, size), crystallite size	*λ* (nm)	*V*-bais (volt.)	Figure of merit			Response time/fall time	Ref.
					*R*_*λ*_ (A W^−1^)	D* (Jones)	EQE (%)		
Hydrothermal, n-CdS/p-Si heterojunction	p-Si (100), thickness 500 µm, resistivity 1–10 Ω cm	Nanorods, 200 nm, 30–35 nm	532	0 (self-powered)	0.00392	7.91 × 10^8^	__	190.8/298.4 µs	39
CdS nanoparticle powder synthesized chemically + deposited by thermal evaporation, Al/CdS-PS : pSi	p-Si, thickness375 ± 25 µm, resistivity 1–10 Ω cm, electrochemical etching (48% HF + ethanol, 22.1 mA cm^−2^ for 30 s)	Flower-like structure with 170 nm layer thickness, 32 nm	400	2	0.6	__	180	0.16/0.35 s	40
Chemical co-precipitation of CdS NPs + drop-dry deposition, CdS/p-Si	p-Si(111), photo-electrochemical etching (HF : ethanol (1 : 1), 2 mA cm^−2^, 30 min)	Spherical, 239.55, 12.93 nm	385	6	0.0007	3.16 × 10^8^	__	__	41
Chemical bath deposition (CBD) Au/CdS/Au	Glass	2D interconnected CdS, 80 ± 10, 12.7 nm	420	10	0.38	2.6 × 10^13^	__	__	42
Pulsed laser deposition (PLD) CdS/Si	p-Si, thickness 525 µm, resistivity 2–11 Ω cm	Spherical, 38–48 nm, 12.59 nm	530	2	48	5.1 ×10^17^	113	112/113 ms	43
Chemical method + drop casting	p-Si(111), resistivity 3–5 Ω cm, electrochemical etching (HF : ethanol, 1 : 1 at 16 mA cm^−2^ for 10 min)	Irregular, 80 nm	577	8.5	1.7	4.6 × 10^11^	__	__	26
CBD, CdS/pyramid-Si	p-Si (100), thickness 500 µm, wet etching (60 wt% KOH at 90 °C for 120 min)	__	__	0 (self-powered)	3.16	__	__	86.4/96.3 µs	44
LACBD + CBD	Glass	Spherical, 5 nm	__	10 V	__	__	__	__	45
Laser ablation in liquid (LAL) + Au pellet ablation (core–shell) + spin coating + IPL annealing	p-Si (111), 500 µm thickness, resistivity 3–5 Ω cm	Spherical, 79–90 nm after IPL. Au–CdS 67–100 nm after IPL. Core–shell morphology	460 520 nm	0 (self-powered)	CdS/Si: 0.19–0.36, Au–CdS/Si: 0.23–0.42	Up to 100% for Au–CdS/Si	__	___	46
LACBD	p-Si (100), thickness500 µm, resistivity 10 Ω cm, (HF : ethanol 1 : 3) for 10 mA cm^−2^ at 8 min	Spherical nanoparticles 11.4 nm	480	3	4.5	1.3 × 10^12^	12	159/208 ms	This study

As shown in Fig. 5(g), no significant deterioration in the response time of the photodetector was observed after several days of storage under laboratory conditions, indicating its good stability. Moreover, the responsivity at the peak wavelength decreased slightly from 4.5 A W^−1^ to 4.35 A W^−1^ after one month of storage, while the responsivity of the photodetector fabricated without laser illumination dropped more notably from 1.9 A W^−1^ to 1.2 A W^−1^ after the same storage period.

**Fig. 6:**
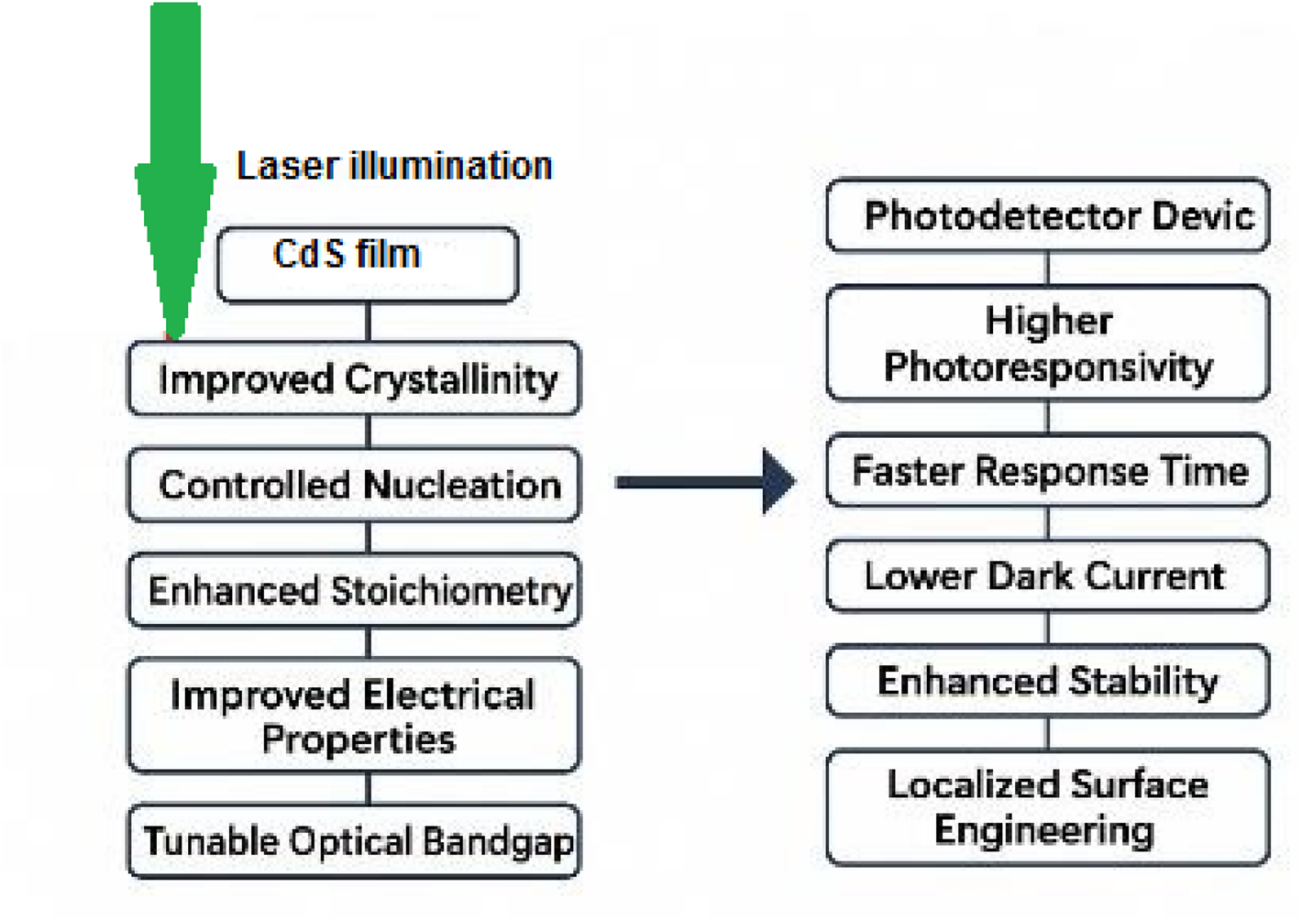
Impact of laser illumination on chemically bath deposited CdS film and CdS/PSi photodetector.

As illustrated in Fig. 6, it can be concluded that employing CW laser illumination during the CBD process of CdS film deposition enhances the film's crystallinity, morphology, and stoichiometry by accelerating thiourea dissociation and promoting the release of S^2−^ ions. This process reduces the density of recombination centers and trap states, thereby facilitating efficient charge carrier transport and lowering the dark current of the photodetector. Consequently, the reduction in defect concentration leads to an overall improvement in the figures of merit and switching properties of the device.

**Fig. 7:**
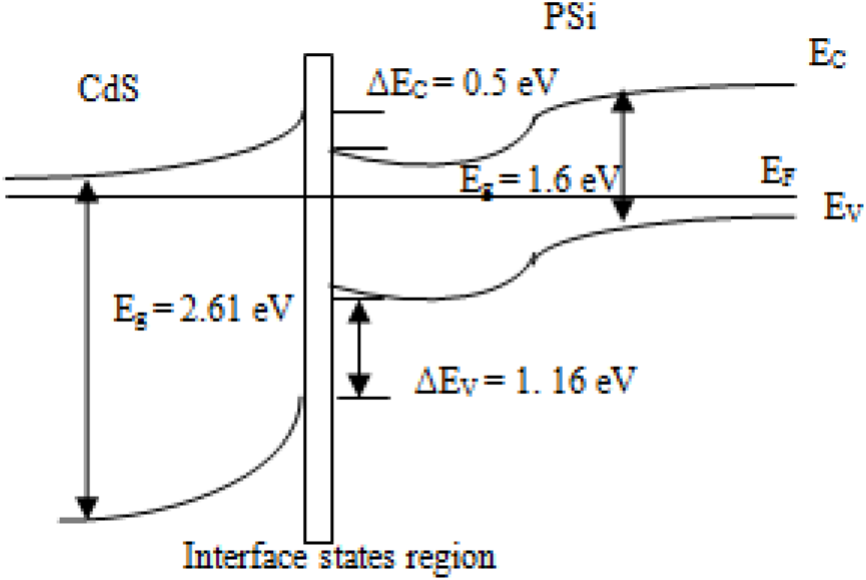
Energy band diagram of CdS/PSi heterojunction photodetector prepared with laser illumination.

The energy band diagram of Cds/PSi heterojunction was established, as shown in Fig. 7. The band offsets Δ*E*_C_ and Δ*E*_V_ were first determined from the following formulas:Δ*E*_C_ = *χ*_Si_ − *χ*_CdS_ = 4.05–4.2 = −0.15 eVΔ*E*_V_ = [*E*_g_(CdS) − *E*_g_(PSi)] − Δ*E*_C_ = 2.61–1.6 + 0.15 = 1.16 eV

This finding means that the small value of Δ*E*_C_ facilitates efficient electron transfer from the CdS film into the porous silicon. In contrast, the relatively large Δ*E*_V_ means that porous silicon creates a barrier that prevents holes from moving from PSi to CdS.

## 4. Conclusions

In this work, we demonstrated a new approach for enhancing the characteristics of CdS films prepared by laser-assisted chemical bath deposition (CBD). The structural and optical characteristics of thin cadmium sulfide (CdS) films were found to be improved when laser illumination used during deposition. X-ray diffraction (XRD) studies show that the CdS film's crystallinity is enhanced when a laser is used prior to deposit. It was discovered that laser illumination enhanced the stoichiometry of the CdS film. The optical absorption findings show that when the laser was employed for the film deposition, the optical band gap of the CdS film increased from 2.38 eV to 2.61 eV. SEM observations confirmed that using of laser illumination resulted in improving the embedding of the CdS grains inside the pores and reduced the grains agglomeration. After using laser illumination, the performance of the CdS/PSi photodetector were enhanced significantly, the photodetector's sensitivity increased from 1.9 to 4.4 A W^−1^ at 480 nm. The ON/OFF current ratio as well as in the rise and fall times were improved. This indicates that employing laser beam illumination during the deposition process is considered an ideal method for preparing CdS films through chemical bath deposition, which produces a film with the necessary characteristics for use as a window in photodetectors.

## Conflicts of interest

The authors declare that they have no known competing financial interests or personal relationships that could have appeared to influence the work reported in this paper.

## Data Availability

The datasets generated during and/or analyzed during the current study are available from the corresponding author on reasonable request.

## References

[cit1] Khallaf H., Oladeji I. O., Chai G., Chow L. (2008). Thin Solid Films.

[cit2] Goesmann H., Feldmann C. (2010). Angew. Chem., Int. Ed..

[cit3] Robel I., Kuno M., Kamat P. V. (2007). J. Am. Chem. Soc..

[cit4] Lee J. H., Lee D. J. (2007). Thin Solid Films.

[cit5] Ikhmayies S. J., Ahmad-Bitar R. N. (2010). Appl. Surf. Sci..

[cit6] Tomakin M., Altunbaş M., Bacaksiz E. M. İ. N., Çelik Ş. (2012). Thin Solid Films.

[cit7] Orlianges J. C., Champeaux C., Dutheil P., Catherinot A., Mejean T. M. (2011). Thin Solid Films.

[cit8] Apolinar-Iribe A., Acosta-Enríquez M. C., Quevedo-Lopez M. A., Ramírez-Bon R., Castillo S. (2010). Chalcogenide Lett..

[cit9] Kitaev G. A., Uritskaya A. A., Mokrushin S. G. (1965). Russ. J. Phys. Chem..

[cit10] Lisco F., Kaminski P. M., Abbas A., Bass K., Bowers J. W., Claudio G., Walls J. M. (2015). Thin Solid Films.

[cit11] Samiyammal P., Parasuraman K., Balu A. R. (2019). Superlattices Microstruct..

[cit12] Sami R., Ghazai A. J. (2022). J. Nanostruct..

[cit13] Ahmed F. M., Muhammed Ali A. M., Ismail R. A., Fakhri M. A., Salim E. T. (2023). J. Mater. Sci.: Mater. Electron..

[cit14] Kakhaki Z. M., Youzbashi A. A., Sangpour P., Naderi N., Orooji Y. (2022). Mater. Sci. Semicond. Process..

[cit15] Eesa M. W. (2016). Iraqi J. Phys..

[cit16] Li Y., Song X. Y., Song Y. L., Ji P. F., Zhou F. Q., Tian M. L., Li X. J. (2016). Mater. Res. Bull..

[cit17] Srinivasarao K., Mohanbabu P., Nagaraju N., Verma S., Rao B. T., Kumar A. A. (2023). Mater. Today: Proc..

[cit18] Naderi N., Hashim M. R. (2012). Int. J. Electrochem. Sci..

[cit19] Hadi H. A., Kasim S. T., Farhan F. K. (2023). Silicon.

[cit20] Tian B., Zheng X., Kempa T. J., Fang Y., Yu N., Yu G., Lieber C. M. (2007). Nature.

[cit21] Wang X., Peng K. Q., Pan X. J., Chen X., Yang Y., Li L., Lee S. T. (2011). Angew. Chem., Int. Ed..

[cit22] Katiyar A. K., Sinha A. K., Manna S., Ray S. K. (2014). ACS Appl. Mater. Interfaces.

[cit23] Canham L. T. , Properties of Porous Silicon, IEE Press, London, 1997

[cit24] Puretzky A. A., Geohegan D. B., Fan X., Pennycook S. J. (2000). Appl. Phys. A.

[cit25] Gou G., Dai G., Qian C., Liu Y., Fu Y., Tian Z., Gao Y. (2016). Nanoscale.

[cit26] Ismail R. A., Khashan K. S., Alwan A. M. (2017). Silicon.

[cit27] Adegoke K. A., Iqbal M., Louis H., Bello O. S. (2019). Mater. Sci. Energy Technol..

[cit28] Al-Hussam A. M., Jassim S. A. J., Assoc J. (2012). Arab Univ. Basic Appl. Sci..

[cit29] Aida M. S. and Hariech S., in Advances in Energy Materials, Springer, Cham, 2020, pp. 49–75, 10.1007/978-3-030-50108-2_3

[cit30] Ismail R. A., Al-Samarai A. M. E., Ali A. Y. (2018). Optik.

[cit31] Scherrer P. , in Kolloidchemie: Ein Lehrbuch, Springer, Berlin, 1912, pp. 387–409, doi: 10.1007/978-3-662-33915-2_7

[cit32] Williamson G. K., Smallman R. E. (1956). Philos. Mag..

[cit33] Subbaiah Y. V., Prathap P., Reddy K. R. (2006). Appl. Surf. Sci..

[cit34] Abdulhameed A. S., Hadi H. A., Ismail R. A. (2024). J. Mater. Sci.: Mater. Electron..

[cit35] Hadi H. A. (2014). J. Fundam. Appl. Sci..

[cit36] Hayif N. D., Hadi H. A., Hashim I. H. (2025). Silicon.

[cit37] Alwazny M. S., Ismail R. A., Salim E. T. (2022). Appl. Phys. A.

[cit38] Shaker S. S., Ismail R. A., Ahmed D. S. (2022). J. Inorg. Organomet. Polym. Mater..

[cit39] Ren Z., Wang Q., Zhang G., Zhang T., Liu J., Wang S., Qiao S. (2023). Surf. Interfaces.

[cit40] Sarmah S., Das M., Sarkar D. (2021). Mater. Today: Proc..

[cit41] Ibnaouf K. H., Firas O. M., Alanazi B. K., Rajamanickam S., Ahmed N. M., Sugumaran S. D., Almessiere M. A. (2025). Sens. Actuators, A.

[cit42] Waldiya M., Narasimman R., Bhagat D., Vankhade D., Mukhopadhyay I. (2019). Mater. Chem. Phys..

[cit43] Salih E. Y. (2024). Opt. Mater..

[cit44] Jiang H., Nie M., Pu Z., Fan J., Liu J., Wang S., Qiao S. (2025). Nano Energy.

[cit45] Garcia L. V., Mendivil M. I., Guillen G. G., Martinez J. A., Krishnan B., Avellaneda D., Shaji S. (2015). Appl. Surf. Sci..

[cit46] Abbas H. F., Ismail R. A., Hamoudi W. K., Mohsin M. H. (2025). RSC Adv..

